# 2,5,7-Trimethyl-3-phenyl­sulfonyl-1-benzofuran

**DOI:** 10.1107/S1600536808008477

**Published:** 2008-04-02

**Authors:** Hong Dae Choi, Pil Ja Seo, Byeng Wha Son, Uk Lee

**Affiliations:** a Department of Chemistry, Dongeui University, San 24 Kaya-dong, Busanjin-gu, Busan 614-714, Republic of Korea; b Department of Chemistry, Pukyong National University, 599-1 Daeyeon 3-dong, Nam-gu, Busan 608-737, Republic of Korea

## Abstract

The title compound, C_17_H_16_O_3_S, was prepared by the oxidation of 2,5,7-trimethyl-3-phenyl­sulfanyl-1-benzofuran with 3-chloro­peroxy­benzoic acid. The phenyl ring exhibits a dihedral angle of 81.16 (4)° with the plane of the benzofuran fragment. The crystal structure is stabilized by π–π inter­actions between the furan and benzene rings of neighbouring mol­ecules [centroid–centroid distance = 3.874 (2) Å] and by C—H⋯π inter­actions between a phenyl H atom of the phenyl­sulfonyl substituent and the furan ring of adjacent mol­ecules. In addition, the crystal structure exhibits intra- and inter­molecular C—H⋯O inter­actions.

## Related literature

For the crystal structures of similar substituted benzofuran compounds, see: Choi *et al.* (2007*a* ▶,*b* ▶).
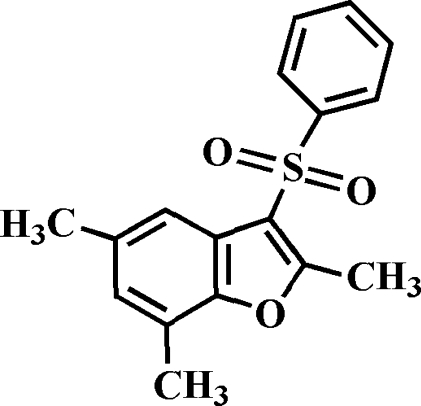


## Experimental

### 

#### Crystal data

C_17_H_16_O_3_S
*M* *_r_* = 300.36
Monoclinic, 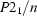

*a* = 9.2468 (7) Å
*b* = 8.4238 (7) Å
*c* = 18.963 (2) Å
β = 91.535 (2)°
*V* = 1476.6 (2) Å^3^
*Z* = 4
Mo *K*α radiation
μ = 0.23 mm^−1^
*T* = 173 (2) K
0.40 × 0.40 × 0.10 mm

#### Data collection

Bruker SMART CCD diffractometer
Absorption correction: none
8704 measured reflections
3212 independent reflections
2544 reflections with *I* > 2σ(*I*)
*R*_int_ = 0.041

#### Refinement

*R*[*F* ^2^ > 2σ(*F* ^2^)] = 0.037
*wR*(*F* ^2^) = 0.104
*S* = 1.03
3212 reflections
193 parameters
H-atom parameters constrained
Δρ_max_ = 0.30 e Å^−3^
Δρ_min_ = −0.38 e Å^−3^

### 

Data collection: *SMART* (Bruker, 2001 ▶); cell refinement: *SAINT* (Bruker, 2001 ▶); data reduction: *SAINT*; program(s) used to solve structure: *SHELXS97* (Sheldrick, 2008 ▶); program(s) used to refine structure: *SHELXL97* (Sheldrick, 2008 ▶); molecular graphics: *ORTEP-3* (Farrugia, 1997 ▶) and *DIAMOND* (Brandenburg, 1998 ▶); software used to prepare material for publication: *SHELXL97*.

## Supplementary Material

Crystal structure: contains datablocks global, I. DOI: 10.1107/S1600536808008477/zl2106sup1.cif

Structure factors: contains datablocks I. DOI: 10.1107/S1600536808008477/zl2106Isup2.hkl

Additional supplementary materials:  crystallographic information; 3D view; checkCIF report

## Figures and Tables

**Table 1:** Hydrogen-bond geometry (Å, °)

*D*—H⋯*A*	*D*—H	H⋯*A*	*D*⋯*A*	*D*—H⋯*A*
C12—H12⋯*Cg*1^i^	0.95	2.81	3.747 (3)	169
C16—H16*C*⋯O3^ii^	0.98	2.48	3.403 (2)	158
C17—H17*A*⋯O2	0.98	2.42	3.135 (3)	129

Symmetry codes: (i) 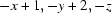; (ii) 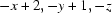. *Cg*1 is the centroid of the O1/C8/C1/C2/C7 furan ring.

**Fig. 1.**
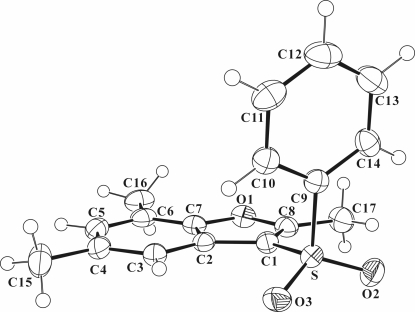
The molecular structure of the title compound, showing displacement ellipsoides drawn at the 50% probability level.

**Fig. 2.**
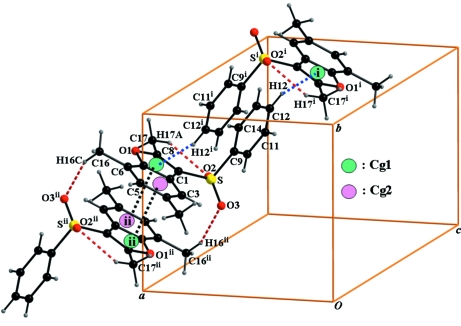
π—π, C—H···π and C—H···O interactions (dotted lines) in the title compound. Cg denotes the ring centroid. [Symmetry code: (i) -x + 1, -y + 2, -z; (ii) -x + 2, -y + 1, -z.]

## supplementary crystallographic information

### Comment

This work is related to earlier communications on the synthesis and structure of substituted benzofuran analogues, *viz*. 2,5-dimethyl-3-methylsulfinyl-1-benzofuran (Choi *et al.*, 2007*a*) and 2,5-dimethyl-3-phenylsulfinyl-1-benzofuran (Choi *et al.*, 2007*b*). Herein we report the molecular and crystal structure of the title compound, 2,5,7-trimethyl-3-phenylsulfonyl-1-benzofuran (Fig. 1).

The benzofuran unit is almost planar, with a mean deviation of 0.009 Å from the least-squares plane defined by the nine constituent atoms. The phenyl ring (C9—C14) is almost perpendicular to the plane of the benzofuran ring system [81.16 (4) °] and is tilted slightly towards it. The crystal packing (Fig. 2) is stabilized by aromatic π—π stacking interactions between the furan and the benzene rings from neighbouring molecules. The *Cg*1···*Cg*2^ii^ distance is 3.874 (2)Å (*Cg*1 and *Cg*2 are the centroids of the O1/C8/C1/C2/C7 furan ring and the C2—C7 benzene ring, respectively, symmetry code as in Fig. 2). The molecular packing is further stabilized by C—H···π interactions between a phenyl H atom of the phenylsulfonyl substituent and the furan ring of the benzofuran unit, with a C12—H12···*Cg*^i^ separation of 2.81 Å (Fig. 2 and Table 1; *Cg*1 is the centroid of the O1/C8/C1/C2/C7 furan ring, symmetry code as in Fig. 2). Additionally, intra- and intermolecular C—H···O interactions in the structure were observed (Fig. 2 and Table 1; symmetry code as in Fig. 2).

### Experimental

3-Chloroperoxybenzoic acid (77%, 471 mg, 2.1 mmol) was added in small portions to a stirred solution of 2,5,7-trimethyl-3-phenylsulfanyl-1-benzofuran (268 mg, 1.0 mmol) in dichloromethane (30 ml) at 273 K. After being stirred for 4 h at room temperature, the mixture was washed with saturated sodium bicarbonate solution and the organic layer was separated, dried over magnesium sulfate, filtered and concentrated in vacuum. The residue was purified by column chromatography (hexane-ethyl acetate, 2:1 *v*/*v*) to afford the title compound as a colorless solid [yield 81%, m.p. 399–400 K; *R*_f_ = 0.61 (hexane-ethyl acetate, 2:1 *v*/*v*)]. Single crystals suitable for X-ray diffraction were prepared by evaporation of a solution of the title compound in benzene at room temperature. Spectroscopic analysis: ^1^H NMR (CDCl_3_, 400 MHz) δ 2.41 (s, 6H), 2.80 (s, 3H), 6.92 (s, 1H), 7.47–7.52 (m, 4H), 8.01 (d, J = 7.68 Hz, 2H); EI—MS 300 [*M*^+^].

### Refinement

All H atoms were positioned geometrically and refined using a riding model, with C—H = 0.95 Å for aromatic H atoms, 0.99 Å for methylene H atoms and 0.98 Å for methyl H atoms, respectively, and with *U*_iso_(H) = 1.2Ueq(C) for aromatic and methylene, *U*_iso_(H) =1.5Ueq(C) for H atoms for methyl H atoms.

### Crystal data

**Table:**

C_17_H_16_O_3_S	*F*_000_ = 632
*M**_r_* = 300.36	*D*_x_ = 1.351 Mg m^−^^3^
Monoclinic, *P*2_1_/*n*	Mo *K*α radiation λ = 0.71073 Å
Hall symbol: -p 2yn	Cell parameters from 4291 reflections
*a* = 9.2468 (7) Å	θ = 2.5–28.3º
*b* = 8.4238 (7) Å	µ = 0.23 mm^−^^1^
*c* = 18.963 (2) Å	*T* = 173 (2) K
β = 91.535 (2)º	Block, colorless
*V* = 1476.6 (2) Å^3^	0.40 × 0.40 × 0.10 mm
*Z* = 4	

### Data collection

**Table:**

Bruker SMART CCD diffractometer	3212 independent reflections
Radiation source: fine-focus sealed tube	2544 reflections with *I* > 2σ(*I*)
Monochromator: graphite	*R*_int_ = 0.041
Detector resolution: 10.0 pixels mm^-1^	θ_max_ = 27.0º
*T* = 173(2) K	θ_min_ = 2.5º
φ and ω scans	*h* = −11→11
Absorption correction: none	*k* = −7→10
8704 measured reflections	*l* = −24→23

### Refinement

**Table:**

Refinement on *F*^2^	Secondary atom site location: difference Fourier map
Least-squares matrix: full	Hydrogen site location: difference Fourier map
*R*[*F*^2^ > 2σ(*F*^2^)] = 0.037	H-atom parameters constrained
*wR*(*F*^2^) = 0.104	*w* = 1/[σ^2^(*F*_o_^2^) + (0.0467*P*)^2^ + 0.6422*P*] where *P* = (*F*_o_^2^ + 2*F*_c_^2^)/3
*S* = 1.04	(Δ/σ)_max_ < 0.001
3212 reflections	Δρ_max_ = 0.30 e Å^−^^3^
193 parameters	Δρ_min_ = −0.38 e Å^−^^3^
Primary atom site location: structure-invariant direct methods	Extinction correction: none

### Special details

**Table:**

Geometry. All e.s.d.'s (except the e.s.d. in the dihedral angle between two l.s. planes) are estimated using the full covariance matrix. The cell e.s.d.'s are taken into account individually in the estimation of e.s.d.'s in distances, angles and torsion angles; correlations between e.s.d.'s in cell parameters are only used when they are defined by crystal symmetry. An approximate (isotropic) treatment of cell e.s.d.'s is used for estimating e.s.d.'s involving l.s. planes.
Refinement. Refinement of *F*^2^ against ALL reflections. The weighted *R*-factor *wR* and goodness of fit *S* are based on *F*^2^, conventional *R*-factors *R* are based on *F*, with *F* set to zero for negative *F*^2^. The threshold expression of *F*^2^ > σ(*F*^2^) is used only for calculating *R*-factors(gt) *etc*. and is not relevant to the choice of reflections for refinement. *R*-factors based on *F*^2^ are statistically about twice as large as those based on *F*, and *R*- factors based on ALL data will be even larger.

### Fractional atomic coordinates and isotropic or equivalent isotropic displacement parameters (Å^2^)

**Table:**

	*x*	*y*	*z*	*U*_iso_*/*U*_eq_	
S	0.71396 (5)	0.59940 (5)	0.11202 (2)	0.02746 (13)	
O1	1.04792 (13)	0.77976 (15)	0.01610 (7)	0.0331 (3)	
O2	0.77401 (15)	0.57958 (16)	0.18201 (6)	0.0386 (3)	
O3	0.63915 (14)	0.46890 (14)	0.07823 (7)	0.0346 (3)	
C1	0.84920 (18)	0.6601 (2)	0.05599 (9)	0.0270 (4)	
C2	0.84265 (18)	0.6499 (2)	−0.02041 (9)	0.0260 (4)	
C3	0.74772 (19)	0.5861 (2)	−0.07118 (9)	0.0290 (4)	
H3	0.6625	0.5321	−0.0579	0.035*	
C4	0.7809 (2)	0.6035 (2)	−0.14184 (9)	0.0330 (4)	
C5	0.9071 (2)	0.6844 (2)	−0.16049 (10)	0.0346 (4)	
H5	0.9266	0.6957	−0.2092	0.042*	
C6	1.00483 (19)	0.7488 (2)	−0.11156 (10)	0.0328 (4)	
C7	0.96763 (19)	0.7270 (2)	−0.04190 (9)	0.0291 (4)	
C8	0.97377 (19)	0.7376 (2)	0.07465 (9)	0.0304 (4)	
C9	0.59363 (18)	0.7624 (2)	0.11293 (9)	0.0263 (4)	
C10	0.48964 (19)	0.7768 (2)	0.05910 (9)	0.0318 (4)	
H10	0.4830	0.7005	0.0223	0.038*	
C11	0.3956 (2)	0.9051 (2)	0.06032 (11)	0.0402 (5)	
H11	0.3232	0.9165	0.0242	0.048*	
C12	0.4063 (2)	1.0159 (2)	0.11345 (12)	0.0450 (5)	
H12	0.3416	1.1035	0.1137	0.054*	
C13	0.5111 (2)	1.0004 (2)	0.16670 (11)	0.0428 (5)	
H13	0.5180	1.0776	0.2032	0.051*	
C14	0.6058 (2)	0.8729 (2)	0.16692 (10)	0.0329 (4)	
H14	0.6777	0.8615	0.2033	0.040*	
C15	0.6791 (2)	0.5378 (3)	−0.19827 (10)	0.0461 (5)	
H15A	0.6497	0.4301	−0.1853	0.069*	
H15B	0.7282	0.5348	−0.2434	0.069*	
H15C	0.5934	0.6058	−0.2027	0.069*	
C16	1.1407 (2)	0.8345 (2)	−0.13129 (12)	0.0438 (5)	
H16A	1.1486	0.9339	−0.1046	0.066*	
H16B	1.1370	0.8581	−0.1819	0.066*	
H16C	1.2249	0.7675	−0.1202	0.066*	
C17	1.0431 (2)	0.7849 (2)	0.14286 (10)	0.0398 (5)	
H17A	0.9787	0.7586	0.1814	0.060*	
H17B	1.0614	0.8994	0.1427	0.060*	
H17C	1.1348	0.7278	0.1495	0.060*	

### Atomic displacement parameters (Å^2^)

**Table:**

	*U*^11^	*U*^22^	*U*^33^	*U*^12^	*U*^13^	*U*^23^
S	0.0317 (2)	0.0259 (2)	0.0248 (2)	0.00101 (17)	0.00055 (16)	0.00232 (17)
O1	0.0278 (6)	0.0313 (7)	0.0401 (7)	−0.0011 (5)	0.0010 (5)	−0.0019 (6)
O2	0.0440 (8)	0.0429 (8)	0.0287 (7)	0.0047 (6)	−0.0027 (6)	0.0058 (6)
O3	0.0403 (7)	0.0252 (6)	0.0383 (7)	−0.0040 (5)	0.0022 (6)	−0.0008 (5)
C1	0.0264 (8)	0.0274 (9)	0.0273 (8)	0.0029 (7)	−0.0003 (6)	−0.0001 (7)
C2	0.0276 (8)	0.0229 (8)	0.0276 (8)	0.0051 (7)	0.0019 (6)	0.0015 (7)
C3	0.0301 (9)	0.0280 (9)	0.0289 (9)	0.0026 (7)	0.0005 (7)	0.0000 (7)
C4	0.0383 (10)	0.0317 (9)	0.0289 (9)	0.0091 (8)	−0.0003 (7)	0.0010 (8)
C5	0.0430 (10)	0.0332 (10)	0.0281 (9)	0.0124 (8)	0.0082 (8)	0.0045 (8)
C6	0.0334 (10)	0.0260 (9)	0.0395 (10)	0.0081 (7)	0.0098 (8)	0.0050 (8)
C7	0.0287 (8)	0.0238 (8)	0.0348 (9)	0.0043 (7)	0.0007 (7)	−0.0003 (7)
C8	0.0294 (9)	0.0274 (9)	0.0343 (9)	0.0047 (7)	−0.0008 (7)	−0.0013 (7)
C9	0.0253 (8)	0.0252 (8)	0.0286 (8)	−0.0030 (7)	0.0045 (7)	0.0023 (7)
C10	0.0320 (9)	0.0308 (9)	0.0324 (9)	−0.0035 (7)	0.0006 (7)	0.0012 (8)
C11	0.0303 (10)	0.0424 (11)	0.0480 (12)	0.0021 (8)	0.0011 (8)	0.0113 (9)
C12	0.0404 (11)	0.0350 (11)	0.0605 (13)	0.0097 (9)	0.0177 (10)	0.0049 (10)
C13	0.0488 (12)	0.0338 (10)	0.0465 (12)	0.0003 (9)	0.0147 (9)	−0.0076 (9)
C14	0.0339 (9)	0.0345 (10)	0.0307 (9)	−0.0040 (8)	0.0066 (7)	−0.0033 (8)
C15	0.0492 (12)	0.0601 (14)	0.0285 (10)	0.0059 (11)	−0.0055 (8)	−0.0026 (9)
C16	0.0401 (11)	0.0361 (11)	0.0561 (13)	0.0031 (9)	0.0161 (10)	0.0097 (9)
C17	0.0359 (10)	0.0416 (11)	0.0413 (11)	0.0006 (9)	−0.0096 (8)	−0.0055 (9)

### Geometric parameters (Å, °)

**Table:**

S—O2	1.4346 (13)	C9—C10	1.389 (2)
S—O3	1.4401 (13)	C10—C11	1.388 (3)
S—C1	1.7394 (17)	C10—H10	0.9500
S—C9	1.7674 (17)	C11—C12	1.375 (3)
O1—C8	1.367 (2)	C11—H11	0.9500
O1—C7	1.384 (2)	C12—C13	1.387 (3)
C1—C8	1.363 (2)	C12—H12	0.9500
C1—C2	1.451 (2)	C13—C14	1.386 (3)
C2—C3	1.393 (2)	C13—H13	0.9500
C2—C7	1.396 (2)	C14—H14	0.9500
C3—C4	1.390 (2)	C15—H15A	0.9800
C3—H3	0.9500	C15—H15B	0.9800
C4—C5	1.405 (3)	C15—H15C	0.9800
C4—C15	1.511 (3)	C16—H16A	0.9800
C5—C6	1.388 (3)	C16—H16B	0.9800
C5—H5	0.9500	C16—H16C	0.9800
C6—C7	1.386 (2)	C17—H17A	0.9800
C6—C16	1.505 (3)	C17—H17B	0.9800
C8—C17	1.483 (2)	C17—H17C	0.9800
C9—C14	1.386 (2)		
			
O2—S—O3	119.48 (8)	C11—C10—C9	118.54 (17)
O2—S—C1	109.43 (8)	C11—C10—H10	120.7
O3—S—C1	107.29 (8)	C9—C10—H10	120.7
O2—S—C9	108.03 (8)	C12—C11—C10	120.53 (19)
O3—S—C9	107.59 (8)	C12—C11—H11	119.7
C1—S—C9	103.94 (8)	C10—C11—H11	119.7
C8—O1—C7	106.96 (13)	C11—C12—C13	120.35 (19)
C8—C1—C2	107.46 (15)	C11—C12—H12	119.8
C8—C1—S	126.78 (14)	C13—C12—H12	119.8
C2—C1—S	125.53 (13)	C14—C13—C12	120.26 (19)
C3—C2—C7	119.27 (16)	C14—C13—H13	119.9
C3—C2—C1	136.18 (16)	C12—C13—H13	119.9
C7—C2—C1	104.55 (15)	C13—C14—C9	118.66 (18)
C4—C3—C2	118.31 (17)	C13—C14—H14	120.7
C4—C3—H3	120.8	C9—C14—H14	120.7
C2—C3—H3	120.8	C4—C15—H15A	109.5
C3—C4—C5	119.98 (17)	C4—C15—H15B	109.5
C3—C4—C15	119.65 (18)	H15A—C15—H15B	109.5
C5—C4—C15	120.36 (17)	C4—C15—H15C	109.5
C6—C5—C4	123.47 (17)	H15A—C15—H15C	109.5
C6—C5—H5	118.3	H15B—C15—H15C	109.5
C4—C5—H5	118.3	C6—C16—H16A	109.5
C7—C6—C5	114.32 (17)	C6—C16—H16B	109.5
C7—C6—C16	122.04 (18)	H16A—C16—H16B	109.5
C5—C6—C16	123.64 (17)	C6—C16—H16C	109.5
O1—C7—C6	124.99 (16)	H16A—C16—H16C	109.5
O1—C7—C2	110.38 (15)	H16B—C16—H16C	109.5
C6—C7—C2	124.63 (17)	C8—C17—H17A	109.5
C1—C8—O1	110.64 (15)	C8—C17—H17B	109.5
C1—C8—C17	134.26 (17)	H17A—C17—H17B	109.5
O1—C8—C17	115.10 (15)	C8—C17—H17C	109.5
C14—C9—C10	121.66 (17)	H17A—C17—H17C	109.5
C14—C9—S	119.35 (14)	H17B—C17—H17C	109.5
C10—C9—S	118.98 (13)		
			
O2—S—C1—C8	−24.52 (19)	C3—C2—C7—O1	−179.24 (14)
O3—S—C1—C8	−155.54 (16)	C1—C2—C7—O1	0.79 (18)
C9—S—C1—C8	90.68 (17)	C3—C2—C7—C6	1.4 (3)
O2—S—C1—C2	161.63 (14)	C1—C2—C7—C6	−178.60 (16)
O3—S—C1—C2	30.61 (17)	C2—C1—C8—O1	0.6 (2)
C9—S—C1—C2	−83.18 (16)	S—C1—C8—O1	−174.20 (12)
C8—C1—C2—C3	179.23 (19)	C2—C1—C8—C17	−179.81 (19)
S—C1—C2—C3	−5.9 (3)	S—C1—C8—C17	5.4 (3)
C8—C1—C2—C7	−0.81 (19)	C7—O1—C8—C1	−0.07 (19)
S—C1—C2—C7	174.03 (13)	C7—O1—C8—C17	−179.78 (15)
C7—C2—C3—C4	−0.7 (2)	O2—S—C9—C14	20.07 (16)
C1—C2—C3—C4	179.30 (18)	O3—S—C9—C14	150.32 (14)
C2—C3—C4—C5	−0.3 (3)	C1—S—C9—C14	−96.11 (15)
C2—C3—C4—C15	−179.10 (17)	O2—S—C9—C10	−160.35 (13)
C3—C4—C5—C6	0.8 (3)	O3—S—C9—C10	−30.10 (15)
C15—C4—C5—C6	179.52 (17)	C1—S—C9—C10	83.47 (15)
C4—C5—C6—C7	−0.1 (3)	C14—C9—C10—C11	−0.5 (3)
C4—C5—C6—C16	179.55 (17)	S—C9—C10—C11	179.95 (13)
C8—O1—C7—C6	178.91 (16)	C9—C10—C11—C12	0.5 (3)
C8—O1—C7—C2	−0.48 (18)	C10—C11—C12—C13	−0.2 (3)
C5—C6—C7—O1	179.76 (15)	C11—C12—C13—C14	−0.1 (3)
C16—C6—C7—O1	0.1 (3)	C12—C13—C14—C9	0.2 (3)
C5—C6—C7—C2	−0.9 (3)	C10—C9—C14—C13	0.1 (3)
C16—C6—C7—C2	179.37 (16)	S—C9—C14—C13	179.70 (14)

### Hydrogen-bond geometry (Å, °)

**Table:**

*D*—H···*A*	*D*—H	H···*A*	*D*···*A*	*D*—H···*A*
C12—H12···Cg1^i^	0.95	2.81	3.747 (3)	169
C16—H16C···O3^ii^	0.98	2.48	3.403 (2)	158
C17—H17A···O2	0.98	2.42	3.135 (3)	129

Symmetry codes: (i) −*x*+1, −*y*+2, −*z*; (ii) −*x*+2, −*y*+1, −*z*.
